# Characterization of responses to flooding and post – flooding recovery in two *Populus deltoides* clones: physiological and biochemical aspects

**DOI:** 10.1186/1753-6561-5-S7-P94

**Published:** 2011-09-13

**Authors:** Maria Emilia Rodriguez, Guillermo Doffo, Fabio Achinelli, Carlos Bartoli, Virginia Luquez

**Affiliations:** 1 Instituto de Fisiología Vegetal (INFIVE), CONICET CCT La Plata – FCAyF UNLP, CC 327, La Plata, Buenos Aires, 1900, Argentina; 2 Instituto de Fisiología Vegetal (INFIVE), CONICET CCT La Plata – FCAyF UNLP, CC 327, La Plata, Buenos Aires, 1900, Argentina. CIC-Buenos Aires

## Background

In Argentina, most poplar plantations are located at the Paraná River Delta. Climate change models indicate an increase of flooding events for the next decades in this area. Therefore, poplar genotypes with a higher flooding tolerance will be needed for the forest activity. Whether this aim is achieved through traditional breeding or biotechnological developments, it will be important to gain a better understanding of poplar responses to flooding at physiological, biochemical and molecular levels.

In a previous work, we characterized the growth and morphological responses of several poplar clones to 35 days of flooding of the root system [1]. From this group, we selected two *Populus deltoides* clones with different growth responses to flooding: Alton and Stoneville 67. In clone Alton, above ground growth is not affected by flooding, while in Stoneville 67 growth is reduced after the second week of flooding. In the present work, our aim was to identify mechanisms underlying the differences in growth responses to flooding between the two clones. We characterized physiological and biochemical responses in roots and leaves, during a flooding period of 28 days, followed by an after flooding period of three weeks.

## Materials and methods

Two *Populus deltoides* clones were used, Alton and Stoneville 67. Plants were obtained from 20 cm length one year old cuttings. The cuttings were planted in 3.5 L pots filled with a mix of topsoil and sand 50:50, and grown in a greenhouse in a totally randomized design; pots were watered daily to field capacity until the start of the treatment. The flooding treatment was started when the plants reached 50 cm of height. Flooding was imposed by covering the pots with tap water up to 5 cm over the surface soil, during 28 days, after this period flooding was ended and pots previously flooded were removed from the water and left to drain.

Growth in height was measured weekly with a graduate stick. Stomatal conductance was measured in the last expanded leaf with a Decagon SC1 porometer. Electrolyte leakage was measured in root tips. Samples were taken from leaves and roots to measure oxidative damage by thiobarbituric acid reactive substances (TBARs) method and enzymatic and non-enzymatic antioxidants. Leaf discs were taken from the last expanded leaf to measure protein content using the Bradford method. This destructive sampling was carried on in the following dates: day 0 (before the start of flooding); 2 weeks after the start of flooding, 4 weeks after the start of flooding; 1 day after the end of flooding, and 1, 2 and 3 weeks after the end of flooding.

## Results and discussion

After 28 days of flooding, growth in height was reduced 14 % in flooded plants of Stoneville 67 compared with non-flooded control plants, but it was not affected in Alton. This was a lasting difference; three weeks after the end of flooding the height of previously flooded Stoneville 67 plants still was 15% lower than the control plants.

Stomatal conductance was reduced by flooding in both clones, but in Stoneville 67 to a greater extent: 81% compared with only 63% in Alton. The recovery of the stomatal conductance to the control levels after flooding was slow and occurred only after two weeks, and was faster in Alton than in Stoneville 67.

Protein soluble content per unit leaf area of the last expanded leaf increased in flooded plants of both clones, in Alton the increase was two fold, and 2.5 times in Stoneville 67, 28 days after the start of flooding. This increase is due to changes in the structure of the leaves of flooded plants; these leaves have a lower area, and a higher number of stomata and epidermal cells than the control leaves per unit area (Rodriguez et al. unpublished results). Likely, these alterations in the leaf structure will have effects in the photosynthetic activity of leaves.

In several species, the sudden exposure of tissues to oxygen after a period of anoxia can cause oxidative stress; this damage is known as post-anoxic injury [2]. We measured electrolyte leakage to estimate membrane damage. Electrolyte leakage was measured in roots, since these were the organs that were submerged and experienced lack of oxygen. The value was higher in Stoneville 67 than in Alton after 28 days of flooding, but there were no differences between clones or treatments 24 h after the end of flooding and thereafter. Since electrolyte leakage is a general measurement of membrane damage, it cannot be attributed solely to oxidative stress. Therefore, we are measuring TBARs content to confirm if this damage is caused by oxidative stress.

As a preliminary conclusion, it seems that clone Alton is less sensitive to flooding than Stoneville 67 because its leaf stomatal conductance and root membrane integrity are less affected by flooding, and its recovery after the end of the stress period is faster.
